# Predicting Response to Medical Treatment in Acromegaly via Granulation Pattern, Expression of Somatostatin Receptors Type 2 and 5 and E-Cadherin

**DOI:** 10.3390/ijms25168663

**Published:** 2024-08-08

**Authors:** Maximilian Cosma Gliga, Laura Chinezu, Ionela Maria Pascanu

**Affiliations:** 1 Doctoral School of Medicine and Pharmacy, George Emil Palade University of Medicine, Pharmacy, Sciences and Technology of Targu Mures, 540142 Targu Mures, Romania; maximilian-cosma.gliga@umfst.ro; 2 Department of Endocrinology, George Emil Palade University of Medicine, Pharmacy, Sciences and Technology of Targu Mures, 540142 Targu Mures, Romania; ionela.pascanu@umfst.ro; 3 Department of Histology, George Emil Palade University of Medicine, Pharmacy, Sciences and Technology of Targu Mures, 540142 Targu Mures, Romania

**Keywords:** acromegaly, somatostatin receptor ligands, immunohistochemistry, biomarkers, treatment response

## Abstract

Resistance to first-generation somatostatin receptor ligand (fgSRL) treatment in acromegaly is common, making the identification of biomarkers that predict fgSRL response a desired goal. We conducted a retrospective analysis on 21 patients with acromegaly who underwent surgery and subsequent pharmacological treatment. Through immunohistochemistry (IHC), we assessed the expression of the somatostatin receptor subtypes SSTR2 and SSTR5, E-Cadherin, and cytokeratin granulation pattern (sparsely or densely). Patients were divided into responders and non-responders based on their biochemical response to fgSRL and/or the newer agent, Pasireotide, or the GH-blocker, Pegvisomant. Patients resistant to fgSRL (n = 12) exhibited lower SSTR2 and E-Cadherin expressions. Sparsely granulated tumors were more frequent in the non-responder group. SSTR2 (*p* = 0.024, r = 0.49) and E-Cadherin (*p* = 0.009, r = 0.64) positively correlated with the Insulin-like Growth Factor 1 (IGF-1) decrease after fgSRL, while SSTR5 (*p* = 0.107, r = −0.37) showed a trend towards negative correlation. SSTR5 positivity seemed to be associated with Pasireotide response, albeit the number of treated patients was too low (n = 4). No IHC markers correlated with Pegvisomant response. Our findings suggest that densely granulated tumors, with positive SSTR2 and E-Cadherin seem to be associated with favorable fgSRL responses. The strongest predictive value of the studied markers was found for E-Cadherin, which seems to surpass even SSTR2.

## 1. Introduction

Acromegaly is a rare endocrine disorder characterized by increased growth hormone (GH) production, which leads to elevated insulin-like growth factor 1 (IGF-1) levels. In the majority of cases, this results from a pituitary GH-producing neuroendocrine tumor (PitNET) [1]. Without effective biochemical control, indicated by normalized IGF-1 levels, patients with acromegaly face a range of disease-related comorbidities, with cardiovascular disease and malignancy being the main contributors of increased mortality [2]. Most guidelines recommend surgery as a first-line treatment, but the surgical cure rate remains unsatisfactory, ranging from 20 to 70% with high variability across different countries and registries [3,4]. Consequently, a substantial proportion of these patients ultimately require pharmacological intervention. First-generation somatostatin receptor ligands (fgSRL) such as Octreotide and Lanreotide are the main agents used. They act by reducing GH production and induce tumor size shrinkage by binding to somatostatin receptors (SSTRs) on the pituitary tumor, with SSTR2 and the SSTR5 being the most prevalent and studied receptor types [5]. The unsatisfactory response to therapy with these agents, which has been reported to occur in over 50% of cases, remains an important challenge, dependent on the biochemical criteria used to define an optimal response [3,6]. Therefore, for patients unresponsive to this treatment, guidelines suggest the introduction of second-line therapies like the GH-receptor-blocking agent, Pegvisomant, or the newer-generation SRL, Pasireotide [4,7].

Increased attention has been given to the research of biomarkers with the potential to predict treatment response and disease aggressiveness in acromegaly. The goal is to transition from the current “trial and error” management to a more patient-tailored, modern, and personalized medicine approach [8]. High presurgical IGF-1 levels, large tumor volume, or T2 MRI hyperintensity are among the classic clinical biomarkers which have been shown to reliably predict a poor pharmacologic treatment response to fgSRL [9,10]. 

Recently, immunohistochemistry (IHC) evaluation of specific molecules on tumor tissue fragments has gained increasing attention for research aimed at providing predictive biomarkers for acromegaly. The cytokeratin (CK) granulation pattern has traditionally divided somatotropinomas into densely granulated (DG) and sparsely granulated (SG) types, with the latter linked to more aggressive behavior and resistance to fgSRLs [11]. Another area of increased interest revolves around the IHC study of SSTR2 and SSTR5 membrane expressions, with these receptors being the main targets of somatostatin receptor ligands. While absent or low SSTR2 expression typically predicts pharmacologic resistance to fgSRL, positive expression does not always correlate with an optimal response [12]. Modifications in other membranous or intracellular signaling pathway molecules may account for the poor treatment response observed in many of these tumors. For instance, the loss of cell adhesion protein E-Cadherin, recognized as a tumor-suppressor molecule in various cancers, has also been found to be a predictor of increased invasiveness and a lack of therapeutic response to fgSRL [13,14]. 

The relationship between SSTR5 and fgSRL response is less clear, but it has been postulated that high expression of this receptor might be linked to a good response to the somatostatin receptor multiligand, Pasireotide, which is known for its affinity for SSTR5. Despite this, data regarding the relationship between these markers and Pasireotide treatment success remain to be elucidated, as the literature data on this topic are scarce and inconsistent [15,16].

Through our study, we aimed to perform a retrospective IHC analysis of patients with acromegaly who underwent transsphenoidal surgery as primary treatment. The primary focus of our research was to assess the potential relationship between the expression levels of SSTR2, SSTR5, E-Cadherin, Ki-67 proliferation index, the cytokeratin (CK) granulation pattern (DG vs. SG), with the biochemical response to fgSRL treatment. Additionally, as a secondary objective, we sought to analyze the role of these markers in predicting response to Pasireotide, as well as to GH-receptor-blocking agent, Pegvisomant.

## 2. Results

In our preliminary patient selection, we included 34 patients with a known biochemical diagnosis of acromegaly and available paraffin-embedded tissue blocks. From this cohort, a number of 21 patients (67%) were treated with fgSRL for at least 6 months, meeting our study’s final inclusion criteria. Among these patients, four were subsequently also treated with Pasireotide, and seven with Pegvisomant due to resistance to fgSRL. Of the included lot, 12 patients (57%) were classified as fgSRL responders (group 1) while the remaining 9 exhibited resistance to fgSRL (group 2).

### 2.1. Demographic, Clinical, Biochemical and Imaging Characteristics 

The baseline demographic characteristics and other clinical and paraclinical variables are presented in Table 1. There was a male predominance in the non-responder group, while the responder group had an equal gender distribution. The age at surgery was slightly higher in the responder group, though this difference was not statistically significant. Only 2 patients had a microadenoma, both of whom were in the responders group. Tumor maximum diameter before surgery was significantly larger in the non-responders group (29.11 mm vs. 19.08mm). All types of tumor extensions (suprasellar, cavernous sinus and optic chiasm) were significantly more frequent in the non-responders group compared to the responders group.

There were no significant differences in the IGF-1 levels at diagnosis (before surgery), or post surgery at fgSRL treatment initiation between the groups. A detectable tumor remnant at post-surgical MRI was significantly more frequent in the non-responder group compared to the responder group (100% vs. 58.33%). 

As expected, the percentage of IGF-1 reduction after 6 months of treatment was higher in the responder group, where it averaged 56.82%, compared to the non-responder group, which had a mean reduction of 7.42%. 

### 2.2. Histologic and IHC Features

IHC analysis confirmed positivity for GH in all tumors, histologically validating the diagnosis of somatotropinoma. Based on the CK granulation pattern, 11 patients (52.4%) exhibited a DG granulated pattern, and 11 patients (52.4%) had a “fibrous body” appearance, suggestive for a SG tumor, while 2 patients had negative CK staining. The differences in IHC markers expressions between the responders and non-responders are presented in Table 2. The prevalence of SG tumors was higher in the non-responder group (77.77% vs. 33%). SSTR2 was expressed in 19 (90%), while SSTR5 was detected in 20 tumors (95%) of our sample. Regarding the SSTR2 expression, patients in the non-responder group exhibited significantly lower Immunoreactivity score (IRS) compared to the responders (5.11 vs. 9.41), while SSTR5 expression showed no significant difference between the groups (9.11 vs. 8.25). The IHC expression of E-Cadherin was also significantly lower in the non-responder group (1.28 vs. 5.30). 

Another important observation was that all patients with a low (IRS < 4) or negative SSTR2 expression were resistant to fgSRL therapy. However, some patients in this group also had maximum SSTR2 positivity (IRS-12) and still remained unresponsive to the treatment. Patients from the non-responder group had higher Ki-67 indexes compared to the responders, though this difference did not reach statistical significance. 

**Figure 1:**
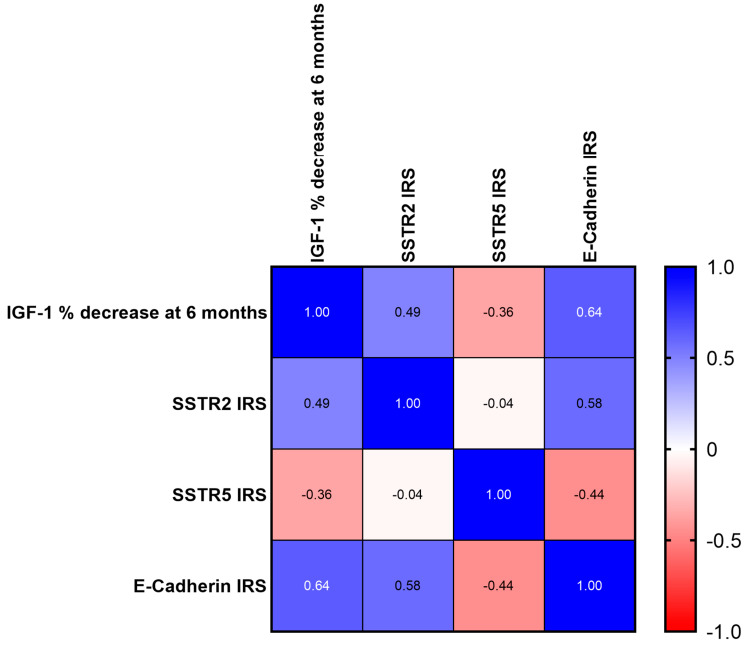
Correlation matrix− IGF−1% decrease after 6 months of fgSRL and IHC markers.

The correlations between IHC markers and IGF-1 decrease at 6 months of fgSRL are illustrated in Figure 1. Notably, we found a strong and positive correlation (r = 0.64, ***p* = 0.009**) between E-Cadherin IHC expression and the percentage of IGF-1 reduction after 6 months of fgSRL therapy. A moderate but significant positive correlation was also observed between SSTR2 expression and the 6-month IGF-1 reduction (r = 0.49, ***p* = 0.024**). In contrast, the correlation for SSTR5 was negative and weak and not statistically significant (r = −0.36, *p* = 0.107). Weak negative correlations were found between SSTR2 and SSTR5 expression without statistical significance, while E-Cadherin positively correlated with SSTR2 (r= 0.58, ***p* = 0.022**) and negatively with SSTR5, though it did not reach statistical significance (r = −0.44, *p* = 0.085). 

**Figure 2:**
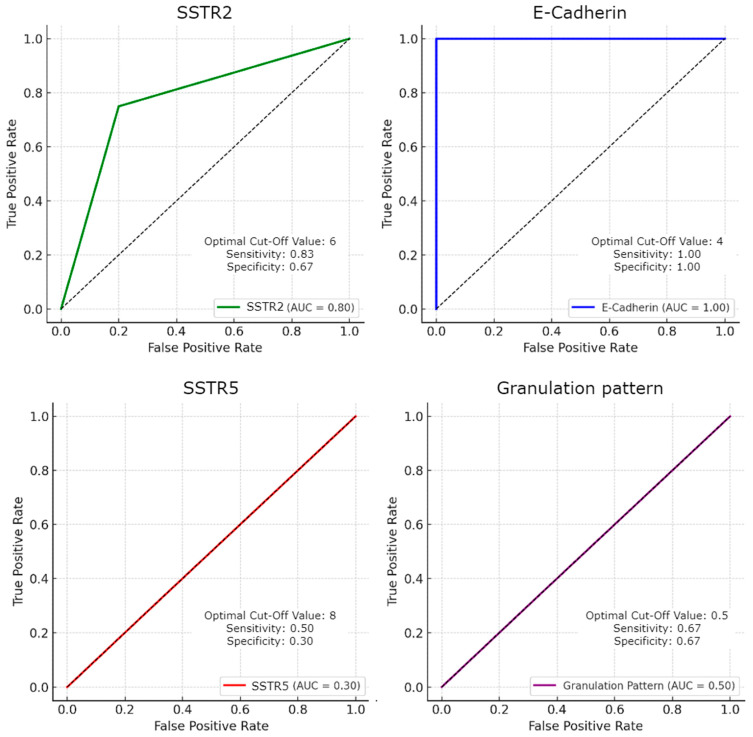
ROC analysis for prediction of fgSRL response—IHC markers: SSTR2, SSTR5, E-Cadherin and Granulation pattern.

A ROC curve analysis was performed to evaluate the predictive value of SSTR2, SSTR5, E-Cadherin, and granulation pattern for fg-SRL treatment response in acromegaly patients, as illustrated in Figure 2. E-Cadherin showed excellent predictive value with an AUC of 1.00 (95% CI: 1.00–1.00), achieving 100% sensitivity and specificity at a cutoff value of 1. SSTR2 demonstrated good predictive ability with an AUC of 0.80 (95% CI: 0.54–1.00), a sensitivity of 77.8%, and specificity of 75.0% at a cutoff value of 7. Conversely, SSTR5 had a poor predictive performance with an AUC of 0.30 (95% CI: 0.08–0.52), a sensitivity of 11.1%, and specificity of 75.0% at a cutoff value of 12. The granulation pattern had no predictive value with an AUC of 0.50 (95% CI: 0.22–0.78).

**Figure 3:**
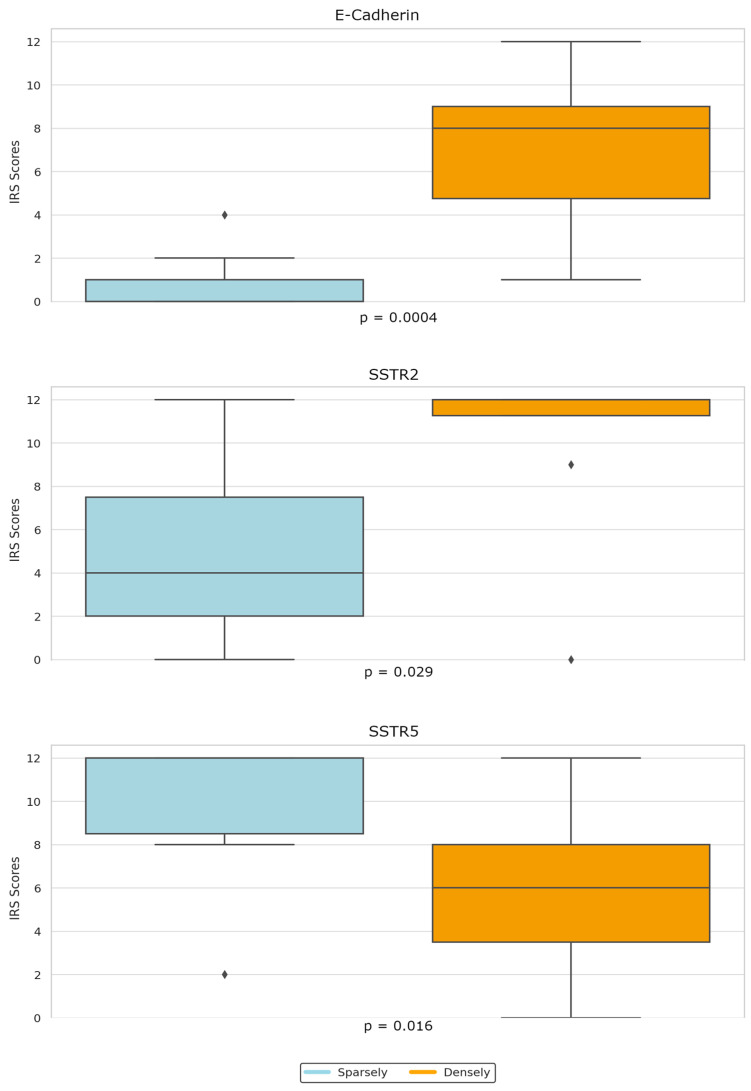
Boxplot graph—Differences in SSTR2, SSTR5 and E-Cadherin IRS score between densely and sparsely granulated tumors.

We also noticed significant differences in the expression of SSTR2, SSTR5, and E-Cadherin between DG and SG tumors. Notably, DG tumors exhibited higher SSTR2 and E-Cadherin scores, whereas SG tumors had significantly higher SSTR5 expression, as illustrated in Figure 3. Furthermore, the percentage of IGF-1 decreased after 6 months of fgSRL treatment was significantly higher for DG tumors compared to the SG ones (54.31% vs. 20.30%). The two patients with negative CK staining responded to fgSRL and both presented high expressions of SSTR2 (IRS: 9 and 12), SSTR5 (IRS 12). 

### 2.3. Second Generation SRL Treatment—Pasireotide

In our patient group, four patients resistant to fgSRL eventually underwent treatment with the second-generation agent —Pasireotide. These patients are presented in Table 3. After 6 months of therapy, two of these patients achieved biochemical control and were classified as responders. One patient achieved partial control (IGF-1 > 1.3× ULN, but a decrease >50% compared to baseline), while another patient remained unresponsive, with poor biochemical control persisting. Except for the non-responder, whose IGF-1 levels increased after 6 months of treatment, the other three patients experienced an IGF-1 decrease between 32.10 and 48.20% compared to baseline values. All patients from this lot had large macroadenomas with suprasellar extension, three having SG tumors, while the non-responder had a DG tumor. Both responders presented a maximum positivity for SSTR5, while the non-responder patient had negative staining for both SSTR5 and SSTR2. The E-Cadherin IRS was very low or negative in all these patients. 

**Figure 4:**
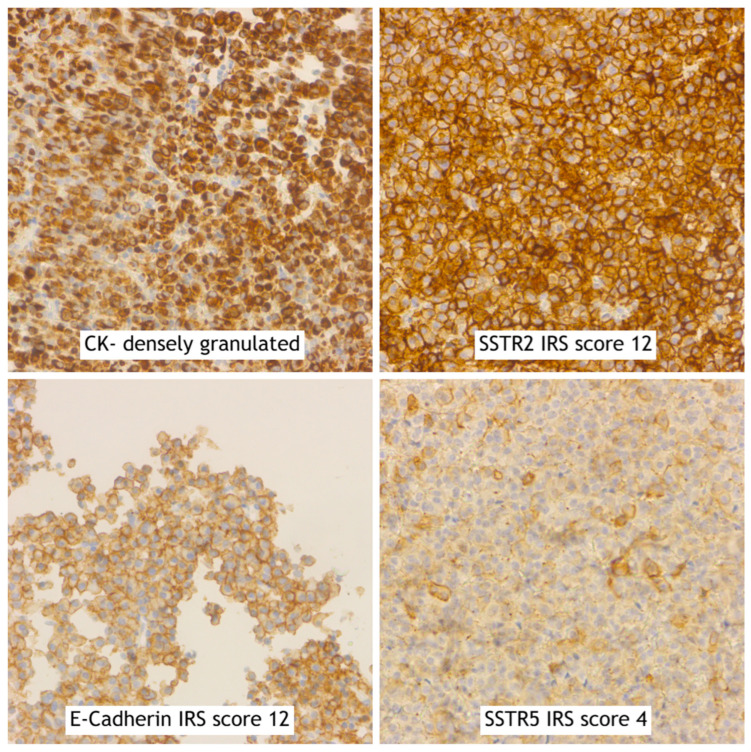
Typical fgSRL responder patient, IHC, 20× magnification.

**Figure 5:**
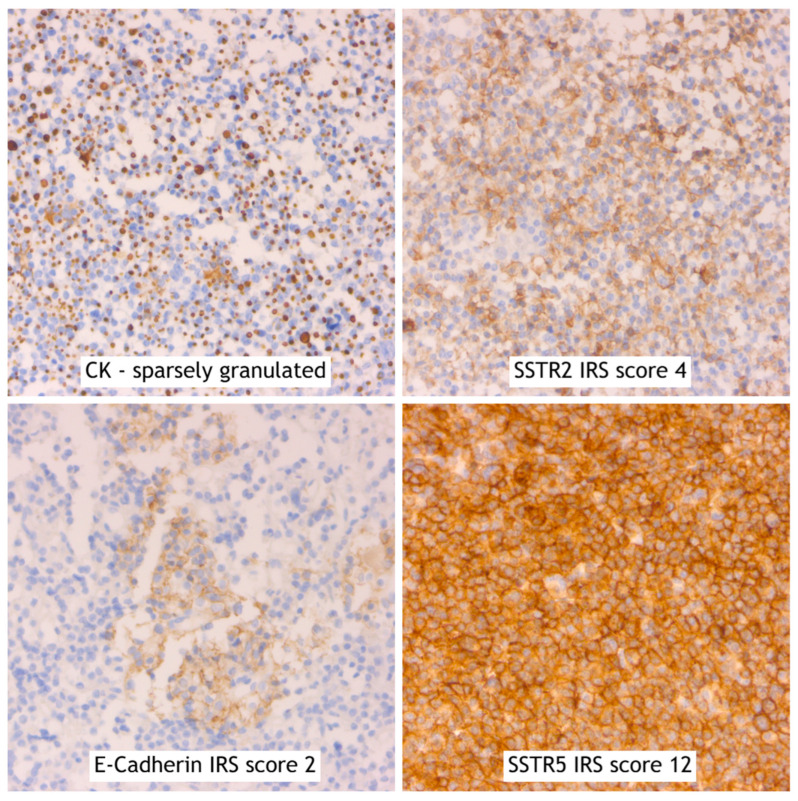
Typical fgSRL non-responder patient, Pasireotide responder, IHC, 20× magnification.

In Figure 4, we present the IHC results with the slides and IRS scores of a typical fgSRL responder patient. The results from a fgSRL-resistant patient and a responder to Pasireotide (Patient 1 from Table 3) are presented in Figure 5.

### 2.4. GH-Blocker Treatment—Pegvisomant

Up to 7 patients in our lot were eventually treated with Pegvisomant. The differences between the patients responsive to Pegvisomant and the non-responders are presented in Table 4. As expected, all these patients were previously deemed resistant to the maximum dose fgSRL therapy. These patients had large invasive macroadenomas, with a mean size of 29.71 mm at diagnosis and there were no significant differences in tumor size between responders and non-responders. After 6 months of therapy, we observed a mean IGF-1% reduction of 49.08%. Among these tumors, five were SG and two were DG, with a mean SSTR2 IRS of 5.71 and a mean SSTR5 IRS of 8.28. The E-Cadherin expression was weak or negative in all these patients. There were no significant differences between the responders and non-responders regarding IHC expression of any of these markers. 

## 3. Discussion

Through our study, we aimed to analyze the predictive value of several IHC markers for treatment response in a sample of patients with acromegaly that underwent medical treatment with fgSRL and/or second line-pharmacological agents, such as Pasireotide or Pegvisomant. All 21 included patients had a persistence of active acromegaly following transsphenoidal surgery as the primary treatment, which imposed the introduction of pharmacologic therapy.

The mean age of the patients was in the fifth decade (46 years old), with younger patients often showing resistance to treatment, a trend also observed in similar studies. For instance, a study by Berton et al. noted younger ages in resistant groups, though not statistically significant, while another study by Luo et al. reported no age differences between resistant and responsive patients. Gender did not significantly influence treatment response in our cohort, consistent with findings from other studies [17,18].

Over 90% of patients had macroadenomas in our sample, which is common in acromegaly due to diagnostic delays. These patients, requiring post-surgical medical treatment, often had larger, difficult to resect tumors. Non-responders to fgSRL typically had larger tumors with more invasive features (suprasellar, cavernous sinus, and optic chiasm extension) and more often detectable tumor remnants post-surgery compared to responders, aligning with findings from other studies suggesting that more extensive tumor resection correlates with better fgSRL treatment outcomes [17,18]. While some studies found higher presurgical IGF-1 levels in fgSRL-resistant patients, our findings did not reveal statistically significant differences, although non-responders had slightly higher IGF-1 mean values [17,18,19,20,21].

Somatotroph tumors are divided into densely granulated (DG) and sparsely granulated (SG) types based on cytokeratin staining patterns. DG tumors, resembling normal somatotroph cells, typically have an indolent behavior, better surgical outcomes, and higher fgSRL response rates, often exhibiting higher SSTR2 positivity [11,22]. Conversely, SG tumors, which are more prevalent in younger patients, are associated with larger, more aggressive tumors, often resistant to fgSRL and showing lower SSTR2 expressions [11,23,24]. In our study, SG tumors were more common (52.4%), likely due to a selection bias as our sample only included patients requiring post-surgical pharmacological treatment, potentially excluding those “cured” surgically with less aggressive DG tumors [23,25]. Similar to the findings by other authors [18,22], we observed that DG tumors had a better response to fgSRL, indicated by a higher IGF-1 decrease after 6 months of treatment. Interestingly, in a study by Dehghani et al., while DG tumors had a higher remission rate after surgery, no significant differences in pharmacologic treatment response were noted between DG and SG tumors [26]. We also found higher SSTR2 and E-Cadherin expression in the DG tumors, while SSTR5 expression was higher in the SG group. These results are in line with findings from other studies for SSTR2 and E-Cadherin, although evidence for SSTR5 is less clear, with some studies reporting higher expressions in the SG tumors and others finding no significant differences among the groups [12,13,27].

SSTR2 is commonly expressed in GH-secreting tumors. We noticed a positive expression in 90% of our sample, aligning with the findings of other studies [28]. SSTR2 expression is a well-known predictor of favorable outcomes after fgSRL therapy, primarily because these agents exert their effects on reducing GH production and causing tumor shrinkage through preferential binding on this SSTR subtype [5,11,29]. As expected, negative IHC expression of SSTR2 typically predicts treatment resistance, a finding confirmed in our study where no patients with an SSTR2 IRS score below three responded to fgSRL. Notably, consistent with similar studies [12], we confirmed the predictive role of SSTR2 for treatment response, with responders having significantly higher SSTR2 expressions, and a significant correlation observed between SSTR2 IRS scores and IGF-1 reduction after 6 months of therapy.

The role of the SSTR5 receptor in driving the response to pharmacological treatment of acromegaly remains less established, as studies have yielded heterogeneous results. We observed a high prevalence of SSTR5 positivity in our sample (95%), unlike other reports where lower positivity rates were noted (60–83%) [27,28]. This discrepancy might be explained by the high prevalence of SG tumors in our cohort, which tend to have higher expression rates of SSTR5. We found no significant differences in SSTR5 levels between responders and non-responders and observed a negative correlation between SSTR5 and E-Cadherin, which was also reported in another study by Venegas-Moreno et al. [13]. Interestingly, some authors found that SSTR5 expression is associated with favorable outcomes to fgSRL only for DG tumors [28]. However, in other studies, similar to our findings, no such associations were observed [10,30]. 

A significant aspect of the SSTR5 receptor is its role in modulating the response to the second-generation SRL agent, Pasireotide. In vitro studies have shown that Pasireotide binds to all SSTR receptors but, unlike fgSRL, possesses a high affinity for the SSTR5 [31]. This drug has been found to be highly effective in reducing IGF-1 in patients uncontrolled by fgSRL in several clinical trials [32,33]. Recent studies have investigated the relationship between Pasireotide efficacy and SSTR5 expression, but results remain controversial, and these studies were performed on small patient samples. In a study by Iacovazzo et al., SSTR5 positivity correlated with higher IGF-1 reductions after Pasireotide treatment, and none of the SSTR5 negative patients responded to Pasireotide [34]. Interestingly, contrasting results were found in the prospective study performed by Muhammad et al., which observed that SSTR2 rather than SSTR5 expression correlated with the response to Pasireotide. Although the number of patients treated with Pasireotide in our study was too low to draw relevant conclusions, we observed that all responders (n = 2) had SG tumors and a maximum IRS of SSTR5, while their SSTR2 expression was low (<4). Conversely, the non-responder patient had a negative SSTR5 expression. These findings, similar to those found in other similar studies, suggest that patients with SG tumors and high SSTR5 expressions might be suitable candidates for direct initiation of Pasireotide treatment.

The cell-adhesion protein, E-Cadherin, is another important IHC marker for determining the behavior of GH-producing tumors. The loss of this protein was linked to epithelial–mesenchymal transition (EMT), a process related to increased invasiveness and therapeutic resistance in different types of tumors [35,36]. In our study, perhaps the most significant finding was that E-Cadherin demonstrated an excellent predictive capability for fgSRL treatment response, with perfect sensitivity and specificity (100%), making it the most reliable marker among those studied, surpassing SSTR2 and the granulation pattern. Additionally, a significant correlation between the E-Cadherin expression and the IGF-1 decrease at 6 months was observed (r = 0.64), which was more pronounced than that of SSTR2 (r = 0.49). Similar to our findings, several other studies have confirmed the strong role of this marker for acromegaly treatment response and behavior [14,30,37], such as the one by Puig-Domingo et al., where they concluded that E-Cadherin might be the best predictor of biochemical response to fgSRL, independent of SSTR2 or histologic subtypes [30]. E-Cadherin expression was found to be lower or negative in SG tumors and positively correlated with SSTR2 [12,13,30]. Conversely, other authors have suggested that E-Cadherin might actually be just a surrogate marker for the granulation subtype and SSTR2, rather than an independent predictor, since it directly correlates with the latter [12]. The molecular mechanisms behind these findings might be related to the relationship of membranous E-Cadherin loss with progression of EMT, which was found to be associated with reduced responsiveness to fgSRL in somatotropinomas [38]. Other molecular mechanisms, such as transcriptional repression or hypermethylation, may underpin the downregulation of E-Cadherin and consequent SSTR2 signaling, leading to pharmacological resistance to fgSRL [35]. The clinical relevance of E-Cadherin in the personalized medicine of acromegaly is also highlighted in the ACROFAST trial, which utilized the IHC expression of this marker, without the addition of SSTR2, to stratify patients into standard or personalized treatment arms. Patients exhibiting positive E-Cadherin expression were treated with standard fgSRL therapy, while those with negative E-Cadherin expression were directed towards a personalized treatment approach, which included Pegvisomant or combination therapy. The trial’s findings suggested that this stratification led to improved outcomes compared to the traditional trial-and-error method, underscoring the effectiveness of tailored treatment approaches based on E-Cadherin status [39]. All these findings advocate for E-Cadherin as a primary biomarker in personalized treatment algorithms for acromegaly.

Another useful marker for assessing the potential for recurrence and invasiveness in tumors is the nuclear antigen Ki-67. Previous reports have found higher levels of this marker in SG tumors and in fgSRL-resistant tumors [40,41,42]. Moreover, Ki-67 was found to be correlated with tumor volume and extrasellar invasiveness in a study by Kasuki et al. [41]. Although only one patient in our cohort had a Ki-67 index greater than 3%, making results hard to interpret, we also observed higher Ki-67 indexes in SG tumors and in the fgSRL-resistant group, although these differences did not reach statistical significance. 

The peripheral-acting GH-receptor antagonist Pegvisomant is a promising option for patients with acromegaly who are unresponsive to other treatment options. According to current guidelines and protocols, most of the patients started on this drug are typically those with poor biochemical control after fgSRL, sometimes using Pegvisomant monotherapy or in combination with other agents [4,7]. There are limited data regarding potential biomarkers for treatment response to this agent. In a study by Chiloiro et al., higher Ki-67 indexes and higher baseline IGF-1 values were observed in Pegvisomant-unresponsive patients [43]. We also noticed slightly higher Ki-67 indexes and larger tumor sizes in the patients unresponsive to this drug, but these differences did not reach statistical significance. As expected, given the peripheral action of this drug, no other IHC markers correlated with Pegvisomant response.

One of the main limitations of our study lies in its retrospective nature, as conducting a prospective IHC study for a rare disease like acromegaly remains challenging. Another limitation is the reduced sample size, which may be due to the stringent inclusion criteria (only patients with pharmacological treatment with fgSRL at least 6 months after surgery). However, we consider the main strength of our study to be the automated IHC procedures with simultaneous evaluation of multiple markers, using the standardized IRS score, which considers both staining intensity and the percentage of positive cells, thereby providing an accurate and reliable assessment for each marker analyzed. 

A potential barrier to the routine implementation of IHC evaluation for these predictive markers for somatotropinomas remains the lack of standardization in the preparation and the methodology/technique used. Furthermore, there is no common, validated set of antibodies, or an approved universal scoring system, with the IRS score being the most commonly used in recent studies. Another limitation could be the costs associated with such techniques, including the required infrastructure, specialized laboratory, trained personnel, and availability of consumables such as the antibodies and associated materials used for IHC preparation and staining. 

## 4. Materials and Methods

### 4.1. Patients

In this multi-centric, retrospective, observational study, we enrolled patients with a confirmed biochemical diagnosis of acromegaly, adhering to the current national guidelines for acromegaly diagnosis and treatment [44]. We examined the medical files and hospital discharge papers to gather the relevant clinical and paraclinical data from the patients’ medical history. These encompassed details such as age and gender at diagnosis, presurgical IGF-1 and GH/GH nadir in the oral glucose tolerance test or GH mean/24 h, presurgical magnetic resonance imaging (MRI) findings such as tumor maximum diameter in millimeters (mm), tumor extension and type (suprasellar, cavernous sinus, optic chiasm), postsurgical IGF-1 levels in evolution, post-surgical MRI findings, pharmacologic treatment history, pituitary function hormonal tests, and information about relevant comorbidities (ophthalmologic, cardiovascular disease, diabetes insipidus, malignancies). All the patients underwent transsphenoidal surgery in three large tertiary centers in Romania between 2005 and 2023. For the current study, we selectively included patients in whom active acromegaly had persisted after surgery and, subsequently, all these patients were initiated on fgSRL treatment with Octreotide long-acting release (LAR) 20 mg every 4 weeks or Lanreotide autogel 120 mg every 56 days. For patients who were unresponsive after 3 months of treatment, doses were increased according to the current national acromegaly treatment protocol to Octreotide LAR 40 mg every 4 weeks and Lanreotide autogel 120 mg every 28 days [44]. Subsequently, some of those considered resistant were transitioned to Pasireotide LAR 40 mg every 4 weeks, with an increase up to the maximum dose of 60 mg every 4 weeks, or Pegvisomant 10–30 mg daily, adjusted based on response. Biochemical response to fgSRL and/or Pasireotide, Pegvisomant, was defined using the baseline IGF-1 levels before the initiation of treatment and the IGF-1 levels after at least 6 months of therapy and a minimum of 3 months with the maximum dose based on the previously mentioned treatment protocol. Patients were categorized into the following two groups: responsive, indicating optimal biochemical control (IGF-1 levels < 1.3× upper limit of normal values [ULN]), and unresponsive, which included both partial responders (decrease in IGF-1 levels > 50% but remaining >1.3× ULN) and non-responders (decrease in IGF-1 levels < 50%). All laboratory tests were performed in tertiary reference centers using chemiluminescence immunoassays. Serum IGF-1 levels (ng/mL) were measured using the LIAISON^®^ IGF-I assay (DiaSorin S.p.A., Saluggia, Italy) according to the manufacturer’s instructions. The intra-assay coefficient of variation (CV) ranged from 2.4 to 5.1%, and the inter-assay CV ranged from 3.8 to 9.6%, with a calibration range of up to 1500 ng/mL and an analytical sensitivity of 3 ng/mL. Results were adjusted for age and gender and expressed as both absolute values and in relation to the upper limit of the normal values of the reference range.

### 4.2. Immunohistochemistry Analysis 

Formalin-fixed paraffin-embedded tissues from patients meeting the inclusion criteria and ensuring sufficient preservation and quality were obtained. These tissues were sectioned, stained with hematoxylin–eosin, and evaluated by an expert pathologist for the presence of tumor tissue. Patients lacking adequate tissue for optimal immunohistochemical analysis were excluded. After an initial selection process and analysis of medical files for data availability on treatment with fgSRL and/or Pasireotide, Pegvisomant, patients fitting all the inclusion criteria were selected for the final IHC analysis. The paraffin-embedded tissue blocks from the patients included in the study underwent a tissue preparation process in a specialized laboratory of the Advanced Medical and Pharmaceutical Research Center of the George Emil Palade University of Medicine, Pharmacy, Science and Technology of Târgu Mureș. All the IHC staining was performed using the BOND-MAX Fully Automated IHC and ISH Staining System. The antibodies were diluted according to the manufacturer’s recommendations for IHC processing or utilized as “ready-to-use”, prediluted formulations. The following antibodies were chosen for IHC staining: Anti-Human Growth Hormone Polyclonal Antibody (Leica Biosystems Cat# PA0727, RRID:AB_10555406), Cytokeratin (CK) 8/18, Clone 5D3 (Leica Biosystems Cat# NCL-5D3-BIOTIN, RRID:AB_876934), used to distinguish between DG and SG granulated tumors; E-Cadherin, Clone 36B5 (Leica Biosystems Cat# NCL-E-Cad, RRID:AB_442084), Recombinant Anti-Somatostatin Receptor 2 Antibody, Clone UMB1—BSA (Abcam Cat# ab134152, RRID:AB_2737601), Recombinant Anti-Somatostatin Receptor 5 Antibody, Clone UMB4 (Abcam Cat# ab109495, RRID:AB_10859946) Ki67, clone-MM (Leica Biosystems Cat# PA0118, RRID:AB_10555423). Due to technical issues with the staining procedure, we were unable to obtain reliable prolactin IHC results.

For the final assessment of SSTR2, SSTR5, and E-Cadherin expressions, we used the Immunoreactivity score, IRS, a scoring system ranging from 0 to 12. The IRS is calculated by multiplying the percentage of stained cells (0–4: 0 = 0% stained cells, 1 ≤ 10%, 2 = 10–50%, 3 = 51–80%, 4 ≥ 80%) with the staining intensity (0 = absent/negative, 1 = weak, 2 = moderate, 3 = strong) [45]. Additionally, based on cytokeratin staining, tumors were categorized as DG-, SG-, or CK-negative. All slides were reviewed by a pituitary specialized expert pathologist blinded to the clinical data, in two separate sessions held more than 4 weeks apart. The final results were expressed as the mean between the two sessions, in order to enhance the reliability and accuracy of the evaluation process. 

### 4.3. Statistical Analysis

For the assessment of normality of the data, we used the Kolmogorov–Smirnov test. Continuous variables, depending on the normality of the distribution, were expressed as means and standard deviations (SD) or medians and interquartile ranges (IQR). For comparison of data groups, we used Mann–Whitney test for non-parametric data and Student’s t-test for parametric data. Chi-squared or Fischer’s exact tests were applied for binary and categorical data. For the correlations of continuous variables, Spearman’s rank-order correlation test order correlation test was performed. Statistical analysis was performed using SPSS software for Windows 22.0 (SPSS, Chicago, IL, USA) and GraphPad Prism version 8.4.3. A significance level of *p* values < 0.05 was considered statistically significant.

## 5. Conclusions

In conclusion, our study highlights the importance of a comprehensive IHC profile encompassing multiple markers in predicting treatment response to various pharmacologic agents in GH-secreting tumors. Consistent with prior research, we reaffirm the strong roles of SSTR2 expression and DG tumor patterns in predicting favorable outcomes to fgSRL. 

While the independent predictive nature of E-Cadherin remains to be fully elucidated, our findings align with previous studies suggesting that it may offer the strongest predictive value for optimal fgSRL treatment response, potentially surpassing the SSTR2 and granulation pattern.

We observed significantly higher SSTR5 positivity in SG tumors, typically associated with lower SSTR2 and E-Cadherin scores, which makes these patients potentially ideal candidates for direct treatment with Pasireotide, the newer, somatostatin receptor multiligand. Conversely, none of the IHC markers studied correlated with response to Pegvisomant, consistent with its peripheral mechanism of action.

Future research involving larger patient cohorts, ideally of a prospective nature, might lead to the validation of these IHC biomarkers for routine clinical application. Additionally, further novel markers could provide further insights into the behavior of these tumors. Finally, integrating these IHC markers into future acromegaly management guidelines will facilitate personalized treatment strategies tailored on each patient’s molecular profile, ultimately improving treatment outcomes, reducing the morbidity and mortality associated with this disease.

## Figures and Tables

**Table 1:** Demographic, clinical, and paraclinical characteristics of the groups.

	All Patients	fgSRL Responders	fgSRL Non-Responders	*p*-Value
Number (%)	21	12 (57%)	9 (43%)	
Gender—Male (%)	9 (43%)	6 (50%)	3 (34%)	0.6605
Gender—Female (%)	12 (57%)	6 (50%)	6 (66%)	0.6605
Age at surgery—years (±SD)	46.14 (±12.89)	48.33 (±12.78)	43.22 (±13.18)	0.3819
Microadenoma (%)	2 (9.52%)	2 (16.66%)	0 (0%)	0.1979
Tumor maximum diameter- at diagnosis -mm (±SD)	23.38 (±11.46)	19.08 (±11.30)	29.11 (±9.158)	0.0438 *
Suprasellar extension (%)	14 (66%)	5 (41.66%)	9 (100%)	0.0071 *
Cavernous sinus extension (%)	10 (47.61%)	3 (25%)	7 (77.77%)	0.0300 *
Optic chiasm compression (%)	5 (23.80%)	0 (0%)	5 (55.55%)	0.0062 *
IGF-1 at diagnosis ng/mL (±SD)	895.3 (±337)	840 (±357)	968 (±313)	0.4011
IGF-1 at diagnosis xULN (±SD)	3.654 (±1.41)	3.315 (±1.51)	4.106 (±1.20)	0.2132
IGF-1 post-surgery at fgSRL initiation ng/mL (±SD)	620.8 (±350.4)	547 (±261.5)	711 (±435.3)	0.3104
IGF-1 post-surgery at fgSRL initiation xULN (±SD)	2.538 (±1.51)	2.151 (±077)	3.012 (±2.04)	0.2130
Percentage (%) of IGF-1 reduction after 6 months (±SD)	35.65 (±32.42)	56.82 (±16.80)	7.42 (±25.92)	<0.001 *
Detectable remnant post-surgery (%)	16 (76%)	7 (58.33%)	9 (100%)	0.0451 *
Remnant size post surgery-mm (±SD)	14.71 (±15.15)	9.500 (±13.82)	21.67 (±14.70)	0.0661

* = *p* < 0.05 (statistically significant).

**Table 2:** Immunohistochemical markers.

	All Patients	fgSRL Responders	fgSRL Non-Responders	*p*-Value
Densely granulated-CK (%)	8 (38%)	6 (50%)	2 (22%)	0.0951
Sparsely granulated-CK (%)	11 (52.4%)	4 (33%)	7 (77.77%)	0.0951
SSTR2 IRS (±SD)	7.57 (±4.64)	9.41 (±4.16)	5.11 (±4.25)	0.0315 *
SSTR5 IRS (±SD)	8.61 (±4.00)	8.25 (±3.46)	9.11 (±4.80)	0.6381
E-Cadherin IRS (±SD)	3.64 (±4.09)	5.30 (±4.37)	1.28 (±2.21)	0.0421 *
Ki-67 (IQR)	0.50 (0.00–1.25)	0.25 (0.00–0.50)	1.50 (0.00–3.00)	0.0841

* = *p* < 0.05 (statistically significant).

**Table 3:** Pasireotide-treated patients.

	Biochemical Response	SSTR2 IRS	SSTR5 IRS	E-Cadherin IRS	Ki-67	Tumor Size- Pre-Surgery (mm)	IGF-1 Decrease −6 Months (%)
Patient 1	Responder	4	12	2	2	26	40.18
Patient 2	Responder	2	12	0	1.5	28	32.10
Patient 3	Partial	2	8	0	2	24	48.20
Patient 4	Non-responder	0	0	1	0	45	−59.10

**Table 4:** Pegvisomant-treated patients.

	All Patients	Responders (n = 4)	Non-Responders (n = 3)	*p* Value
Tumor size- mm Before surgery (±SD)	29.71 (±9.10)	27.25 (±8.61)	33 (±10.44)	0.1790
IGF-1 decrease after 6 months (%)	49.08 (±28.34)	56.15 (±32.88)	39.65 (±23.56)	0.4967
Densely granulated (%)	2 (28%)	1 (25%)	1 (33.33%)	>0.999
Sparsely granulated (%)	5 (72%)	3 (75%)	2 (66.66%)	>0.999
SSTR2 IRS (±SD)	5.71 (±4.68)	6.00 (±4.32)	5.33 (±6.11)	0.8000
SSTR5 IRS (±SD)	8.28 (±5.21)	8.50 (±4.72)	8.00 (±6.92)	>0.999
E-Cadherin IRS	1.28 (±2.21)	1.50 (±3.00)	1.00 (±1.00)	0.6571
Ki-67 (±SD)	1.78 (±2.48)	0.87 (±1.03)	3.00 (±3.60)	0.3010

## Data Availability

The data used to support the findings of this study are available from the corresponding author upon request.
